# Book Notices

**Published:** 1868-12-15

**Authors:** 


### *BOOK NOTICES.*

The Pharmacist. — We have received the second number of this periodical, and take pleasure in congratulating the Chicago College of Pharmacy, on the very creditable appearance presented by its organ. The Pharmaceutists of Chicago deserve already a high place in the ranks of their profession, and the organization of their College, and the establishment of their Journal, are movements in the right direction toward maintaining and increasing their excellence. The present number contains several interesting articles, which will well repay the reader, particularly those upon Prussic Acid, upon Carbolic Acid, and the comments of the "British Medical Journal," upon a "uniform principle of prescribing and dispensing."

The latter article contains some, not unmerited, strictures upon the lack of system, not to say carelessness, of physicians in prescribing, which might be profitably applied by many members of the medical profession. While acknowledging the justice of these comments, we take occasion, in this connection, to call the attention of the College of Pharmacy to a practice prevalent amongst certain self-styled pharmacists in this city, of dispensing medicines in packages bearing neither the name of the physician prescribing, nor the druggist dispensing them, nor even the patient for whom they were prepared. This has occurred twice, within a very short time, under the observation of the writer. The preparation ordered in the first instance, being a lotion, was received by the patient in a vial having, as a distinctive mark, a piece of wrapping paper, bearing upon its face a number in pencil. The lotion proved entirely inadequate to accomplish its purpose, and was replaced by a repetition of the formula, prepared by Mr. Mill, having a totally different appearance, and effecting promptly the desired result. In the second instance referred to, the formula prescribed contained *Extract of Belladonna.* The preparation was received in a box, bearing a penciled number, but no other distinctive mark whatever. In this instance, also, the drug was inert, and had to be replaced as before. Under the circumstances, in case of accident, it would have been somewhat difficult to identify the offender, and we hope that the College of Pharmacy will redouble its efforts to purge its professional ranks from such reckless tamperers with human life. W. H.

